# Tissue ontogeny and chemical composition influence bacterial biodiversity in the wood and shoot tip of *Populus nigra*

**DOI:** 10.1111/plb.13724

**Published:** 2024-10-02

**Authors:** T. Bose, T. G. Mahomed, K. C. Mbatha, J. C. Joubert, A. Hammerbacher

**Affiliations:** ^1^ Department of Biochemistry, Genetics and Microbiology Forestry and Agricultural Biotechnology Institute (FABI), University of Pretoria Pretoria South Africa; ^2^ Department of Zoology and Entomology Forestry and Agricultural Biotechnology Institute (FABI), University of Pretoria Pretoria South Africa; ^3^ Department of Plant and Soil Sciences Forestry and Agricultural Biotechnology Institute (FABI), University of Pretoria Pretoria South Africa

**Keywords:** 16S rDNA, black poplar, functional annotation, phyllobiome, plant metabolites

## Abstract

- Plant–microbe interactions significantly influence plant growth dynamics and adaptability. This study explores the impact of metabolites on microbial biodiversity in shoot tips and wood of *Populus nigra* under greenhouse conditions, using high‐throughput sequencing and metabolite profiling.
- Branches from *P. nigra* were harvested, rooted, and transplanted into pots for growth. After 3 months, tissue samples from shoot tips and wood were collected, and metabolites extracted and analysed using GC‐MS and LC‐MS. Genomic DNA was extracted and subjected to high‐throughput sequencing for bacterial biodiversity profiling. Both datasets were analysed using bioinformatic and statistical pipelines.
- Metabolite profiling indicated that shoot tips had a higher relative abundance of primary and secondary metabolites, including sugars, fatty acids, organic acids, phenolic acid derivatives and salicinoids, while wood was enriched in flavonoids. Bacterial biodiversity also differed significantly between these tissues, with Clostridiales, Bacteroidales and Bacillales dominating in shoot tips, associated with rapid growth and anaerobic fermentation, while wood tissues were characterized by diazotrophs from Rhizobiales, Sphingomonadales and Frankiales. PCoA clustering confirmed tissue‐specific microbial differences. Functional analysis revealed an enrichment of fundamental cellular processes in shoot tips, while wood exhibited pathways related to degradation and mortality.
- Metabolite profiling revealed significant variations in primary and secondary metabolites, highlighting their influence on microbial biodiversity across plant tissues. The dominance of specific bacterial orders and distinct functional pathways in each tissue suggests a tailored microbial response to the unique environments of shoot tips and wood.

## Graphical abstract

Plant metabolites shape bacterial biodiversity in *Populus nigra* wood and shoot tips.

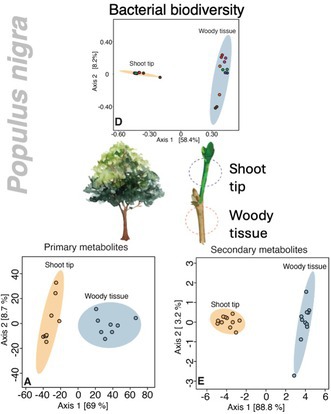

## INTRODUCTION

Plant microbiomes are broadly divided into two categories: the rhizobiome and the phyllobiome. The rhizobiome consists of roots and the soil surrounding them, whereas the phyllobiome consists of leaves, stems, and reproductive organs (Turner *et al*. 2013). In comparison to the rhizobiome, the phyllobiome is nutrient‐poor. Concurrently, the phyllobiome is a more dynamic ecosystem than the rhizobiome, with resident microbes exposed to significant temperature, moisture, and radiation fluxes (Vorholt 2012; Turner *et al*. 2013; Liu *et al*. 2020). Therefore, the microbial biodiversity of the phyllobiome differs significantly among the aerial organs of the plant (Turner *et al*. 2013; Liu *et al*. 2020). For example, the microbes associated with leaves differ remarkably between veins, blades, hairs, and stomata (Lindow & Brandl 2003).

The phyllobiome is colonized by a wide range of microbes (Vorholt 2012); among which are bacteria, fungi, yeast, protists, algae, and phages (Newton *et al*. 2010; Balogh *et al*. 2018; Zhu *et al*. 2018). The microbial community of the phyllobiome is linked to plant health and ontogeny (Leveau 2019). Environmental pressures, host genotype and phenotype, soil geochemistry, and host resources all shape the phyllobiome (Lindow & Brandl 2003; Kembel *et al*. 2014; Leveau 2015; Leveau 2019; Pang *et al*. 2021).

Plant metabolites can be classified as either primary or secondary. Examples of primary metabolites are sugars, amino acids, vitamins, and hormones. Secondary metabolites, which include phenolics, benzoxazinoids, terpenes, and alkaloids, are chemical substances produced by the plant cell through pathways derived from primary metabolic processes (Kossel 1891). These metabolites have distinct and overlapping functions in plants, including growth, stress adaptation, defence, and mediators of organismal interactions (Weng *et al*. 2012; Lebeis *et al*. 2015; Zaynab *et al*. 2019; Erb & Kliebenstein 2020; Jacoby *et al*. 2021; Pang *et al*. 2021). On the other hand, plant microbiomes also play a role in many of the aforementioned activities, directly or indirectly, through regulating plant metabolism. Plants may influence their microbiome by secreting different metabolites, and the microbes, in turn, can influence the metabolome of their host plant (Cotton *et al*. 2019; Huang *et al*. 2019; Voges *et al*. 2019; Chen *et al*. 2019a, 2019b; Jacoby *et al*. 2021). The allocation, diversity, and function of these metabolites, on the other hand, change between plant organs and development phases (Erb & Kliebenstein 2020; Pang *et al*. 2021).

The activity of apical meristems, which are actively dividing cells present in both roots and shoots, shapes the plants. Anatomy of the wood and the constantly differentiating apex in trees are anatomically and metabolically distinct (Evert 2006; Schmitt *et al*. 2021). Photosynthates, for example, are translocated to active growth regions where sugars are needed to sustain new tissue formation in the apex (Lemoine *et al*. 2013; Taiz *et al*. 2015). Other biochemical components linked with these distinct regions, such as secondary metabolites, are often synthesized in the growing shoot apex to protect the nutrient‐rich differentiating tissues (Theis & Lerdau 2003; Li *et al*. 2020). The aggregate of these differences will undoubtedly influence the microbial biodiversity associated with these two regions (Leff *et al*. 2015; Schlechter *et al*. 2019; Mina *et al*. 2020).

Many recent investigations have found that metabolites play an important role in regulating biodiversity and microbial abundance in the phytobiome. Most of these investigations, however, focused on the rhizobiome and were primarily limited to the model plant *Arabidopsis thaliana* (Lebeis *et al*. 2015; Huang *et al*. 2019; Voges *et al*. 2019; Chen *et al*. 2019a, 2019b; Brachi *et al*. 2022). In cases where the phyllobiome was considered, the focal organs were the leaves (Leveau 2019; Liu *et al*. 2020). Therefore, in this study, we chose *Populus nigra* (black poplar), which is widely regarded as the model plant for studying the biology of woody perennials. Poplar has also been used for investigating a wide range of plant–microbe interactions (Ulrich *et al*. 2008; Taghavi *et al*. 2009; Gottel *et al*. 2011; Hur *et al*. 2011; Shakya *et al*. 2013; Beckers *et al*. 2016a; Beckers *et al*. 2016b). We compared the metabolite profiles and bacterial biodiversity associated with two regions of the same poplar sapling, the woody basal part, and the green tips. We hypothesized that: (i) the metabolite profiles of the woody and green tissues of *P. nigra* would differ significantly. The tissue near the shoot tip is expected to be enriched with primary metabolites, such as various sugars and fatty acids to support new tissue formation at the apex, along with secondary metabolites to defend against microbial growth; and (ii) these differences in metabolite profiles will substantially influence the bacterial biodiversity, community composition, and functional metabolic pathways between the two tissue types.

## MATERIAL AND METHODS

### Collection and processing of plant samples

**Fig. 1:**
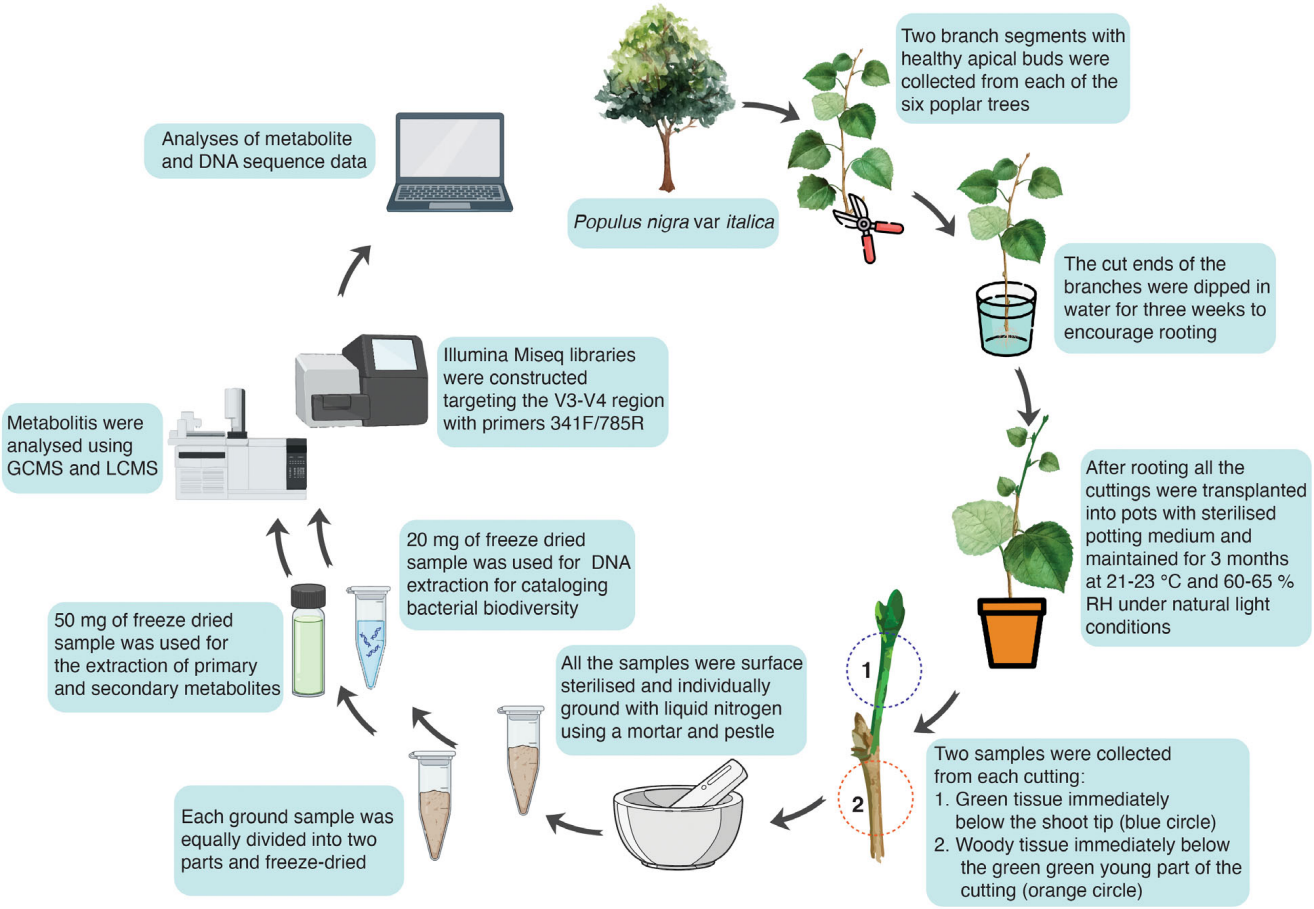
Schematic overview of the study, conducted to determine the influence of plant metabolites and tissue ontogeny on the bacterial biodiversity associated with the branches of *Populus nigra*.

In November 2019, branches from six *P. nigra* var. *italica* trees were collected from the Hillcrest campus of the University of Pretoria (25°44′44.825″ S, 28°15′ 0.058″ E). Six trees were situated adjacent to each other, with a distance of ~10 m between them. Two branch segments were collected from each tree, each measuring 25 cm. Each branch was about 1 cm in diameter and had a healthy apical bud (Fig. 1). All the branches were first rinsed under running tap water, followed by sterile distilled water. Thereafter, the clipped ends of the branches were immersed in distilled water to allow the branches to form roots (Fig. 1). The water in all containers was renewed each week. Following this, all rooted branches were individually transplanted into 12.5 cm plastic pots containing sterile potting soil (Culterra, South Africa). These cuttings were maintained at the plant growth facility of the Forestry and Agricultural Biotechnology Institute (FABI) under natural light conditions. The mean temperature and relative humidity were maintained at 21–23 °C and 60–65%, respectively.

Three months after potting, two samples were collected from each cutting. A 10‐cm section immediately below the apex of the cutting, and another 10‐cm segment below the junction of the woody and young green part of the cutting (Fig. 1). All samples were surface sterilized with 75% ethanol for 1 min, then washed thrice with sterile distilled water, and dried with paper towels. Following this, all the samples were flash‐frozen using liquid nitrogen and stored at −80 °C.

All 24 plant samples (12 branches × 2 sample types) were individually homogenized with a mortar and pestle and liquid nitrogen (Fig. 1). Between samples, the mortar and pestle were surface sterilized with a 2% sodium hypochlorite (NaOCl) solution for 1 min, then rinsed twice with sterile water and dried with a blow dryer. The ground samples were divided into two equal parts and freeze‐dried in 2 ml polypropylene tubes. For each sample, one tube was used for the extraction of genomic DNA, while the other was used for metabolite profiling (Fig. 1).

### Extraction of primary and secondary metabolites from *P. nigra* tissues

Metabolites from each sample were extracted by adding 1.4 ml 80% HPLC grade methanol (Sigma‐Aldrich, St. Louis, MO, USA) amended with 0.4 μl·ml^−1^ of the internal standard, 2‐phenylethanol (Sigma‐Aldrich) to 50 mg of freeze‐dried tissue in a screw cap glass vial. All the samples were then agitated for 2 h at room temperature using benchtop shakers at 200 rpm. Thereafter, all the samples were centrifuged for 1 h at 14,000 rpm. For each sample, 1.2 ml of the supernatant were transferred to a new 2 ml glass screw‐cap vial.

### Biochemical and statistical analysis of metabolite data

#### Primary metabolites

A 1 ml aliquot of methanol extract was dried under constant airflow at 38 °C and resuspended in 100 μl pyridine (Sigma‐Aldrich, Darmstadt, Germany) containing 20 mg·ml^−1^ methoxamine HCl (Sigma‐Aldrich, Buchs, Switzerland) and incubated at 30 °C for 90 min, followed by centrifugation at 1200 rpm for 20 min. The sample was then silylated using 30 μl *N*‐methyl‐*N*‐(trimethylsilyl)trifluoroacetamide. The silylated samples were analysed on an Agilent 7890 gas chromatography–mass spectrometry system (Agilent, Santa Clara, CA, USA) using the methods described in Joubert *et al*. (2023). Metabolites were identified based on their mass spectra, retention times and comparison to pure standards (Joubert *et al*. 2023).

#### Secondary metabolites

Semi‐polar metabolites were analysed using a Bruker Daltronics Esquire 2000 ion trap mass spectrometer (Bruker Daltronics, Germany) coupled to an Agilent 1100 high‐pressure liquid chromatograph. A 1 μl subsample of each sample was injected into a Nucleodur Sphinx RP18ec column, with dimensions of 250 × 4.6 mm and a particle size of 5 μm (Macherey Nagel, Germany). Mobile phases A and B were 0.2% (v/v) formic acid and acetonitrile. A 35 min gradient was used, starting with 0–5 min, 95% A; 5–25 min, 0–65% B in A; 25–28 min 100% B; and 28–35 min 100% A. The mass spectrometer was operated as described in Hammerbacher *et al*. (2014). The major phenolic metabolites were tentatively identified based on their retention times, mass spectra, and published data (Boeckler *et al*. 2011; Boeckler *et al*. 2013; Paetz *et al*. 2016; Ullah *et al*. 2019).

#### Statistical analyses of metabolite data

The chromatograms were processed using Multigroup statistical analysis on the XCMS online platform (Tautenhahn *et al*. 2012). The chromatograms were grouped by tissue ontogeny. Files were managed with ProteoWizard (http://proteowizard.sourceforge.net/) software. The XCMS online retention time‐corrected data table comprising ion intensity values was extracted and statistically analysed using the online platform MetaboAnalyst v5.0 (Pang *et al*. 2022) and R (R Core Team 2022). Only data of tentatively identified metabolites were included in the final datasets. Both datasets were log‐transformed to achieve normality and uniformity of variances before statistical analysis. Principal components analysis was conducted using the default parameters in MetaboAnalyst, and statistical differences between metabolites from different tissue samples were determined using a two‐tailed *t*‐test.

### Extraction of DNA from *P. nigra* tissues and high‐throughput sequencing

The NucleoSpin Plant II kit (Macherey‐Nagel, Germany) was used to extract total genomic DNA from 20 mg freeze‐dried plant tissue following the manufacturer's instructions. The presence of bacterial DNA in each sample was confirmed by PCR amplification of the V3‐V4 hypervariable region of the bacterial 16S rRNA gene using the primers 341F (5′–CCTACGGGNGGCWGCAG–3′) and 785R (5′–GACTACHVGGGTATCTAATCC–3′) (Klindworth *et al*. 2012). Thereafter, a NanoDrop™ One spectrophotometer was used to quantify the concentration of DNA in each sample. These 24 samples were sent to Inqaba Biotechnical Industries (Pty) Ltd, South Africa, for the construction of bacterial amplicon libraries targeting the aforementioned gene region. The Illumina MiSeq platform was used to sequence these libraries. The raw sequencing data were deposited in the NCBI Sequence Read Archive under the accession number PRJNA925423.

### Analyses of bacterial biodiversity data

#### Bioinformatic analysis of bacterial biodiversity data

Paired‐end high‐throughput sequencing data was demultiplexed by the sequencing facility. These paired‐end reads were merged using BBMerge v38.97 (Bushnell *et al*. 2017). These merged data files were further analysed using Quantitative Insights into Microbial Ecology 2 (QIIME 2) v2020.8 (Bolyen *et al*. 2019). The “q2‐dada2” package (Callahan *et al*. 2016) was used for filtering, trimming, denoising, and deleting singletons and chimaeras. During this step, the filtering settings were set to a Phred quality score of 30 and a sequence length limit of 250 bp. All sequences that did not meet these criteria were discarded from the analysis. The “q2‐vsearch” package (Rognes *et al*. 2016) was used for the *de novo* assembly of the reads with 100% sequence similarity. The “qiime feature‐classifier” (Bokulich *et al*. 2018) was used to assign taxonomy to the operational taxonomic units (OTUs) using a SILVA 138 SSURef NR99 bacterial database (Quast *et al*. 2013) trained using the primers 341F and 785R. OTUs with mitochondrial and chloroplast origins were filtered from the final taxonomic table.

#### Statistical analyses of bacterial biodiversity data

Order was used as the preferred taxonomic level for all analyses. The taxonomic heat tree was constructed using the Metacoder (Foster *et al*. 2017). The remaining analyses of the data were conducted using the MicrobiomeAnalyst v2.0 (Dhariwal *et al*. 2017; Chong *et al*. 2020). Low‐count features were filtered based on the mean abundance value, with the minimum count set at 4, while the low‐variance features were removed based on the interquartile range. The remaining features were normalized using total sum scaling (TSS). Alpha diversity was calculated using the Shannon and Simpson indices. Species richness was calculated using the Chao1. For the beta diversity, a PCoA was constructed using the Bray–Curtis index, with PERMANOVA as the statistical method. The core microbiome analysis was for each sample type (shoot tip and woody tissue) with a sample prevalence of 20 and a relative abundance of 0.01.

### Functional annotation of bacterial biodiversity data

PICRUSt2 (Douglas *et al*. 2020) was used to predict the functional abundances of bacterial OTUs identified in shoot tips and woody tissues, against the Kyoto Encyclopaedia of Genes and Genomes (KEGG) Ortholog (KO) database. The OTU table was normalized based on the predicted 16S copy number for each organism in the OTU table. Subsequently, the KOs were grouped by their functions to condense all individual KOs into their corresponding KEGG pathways. To calculate significant distinctions in functional content between the two sample types, White's non‐parametric test with Benjamin‐Hochberg FDR (false discovery rate) adjustment was implemented in the Statistical Analysis of Metagenomic Profiles (STAMP) software (Parks *et al*. 2014), and the outcomes were visualized using an extended error bar plot.

## RESULTS

### Biochemical and statistical analysis of metabolite data

**Fig. 2:**
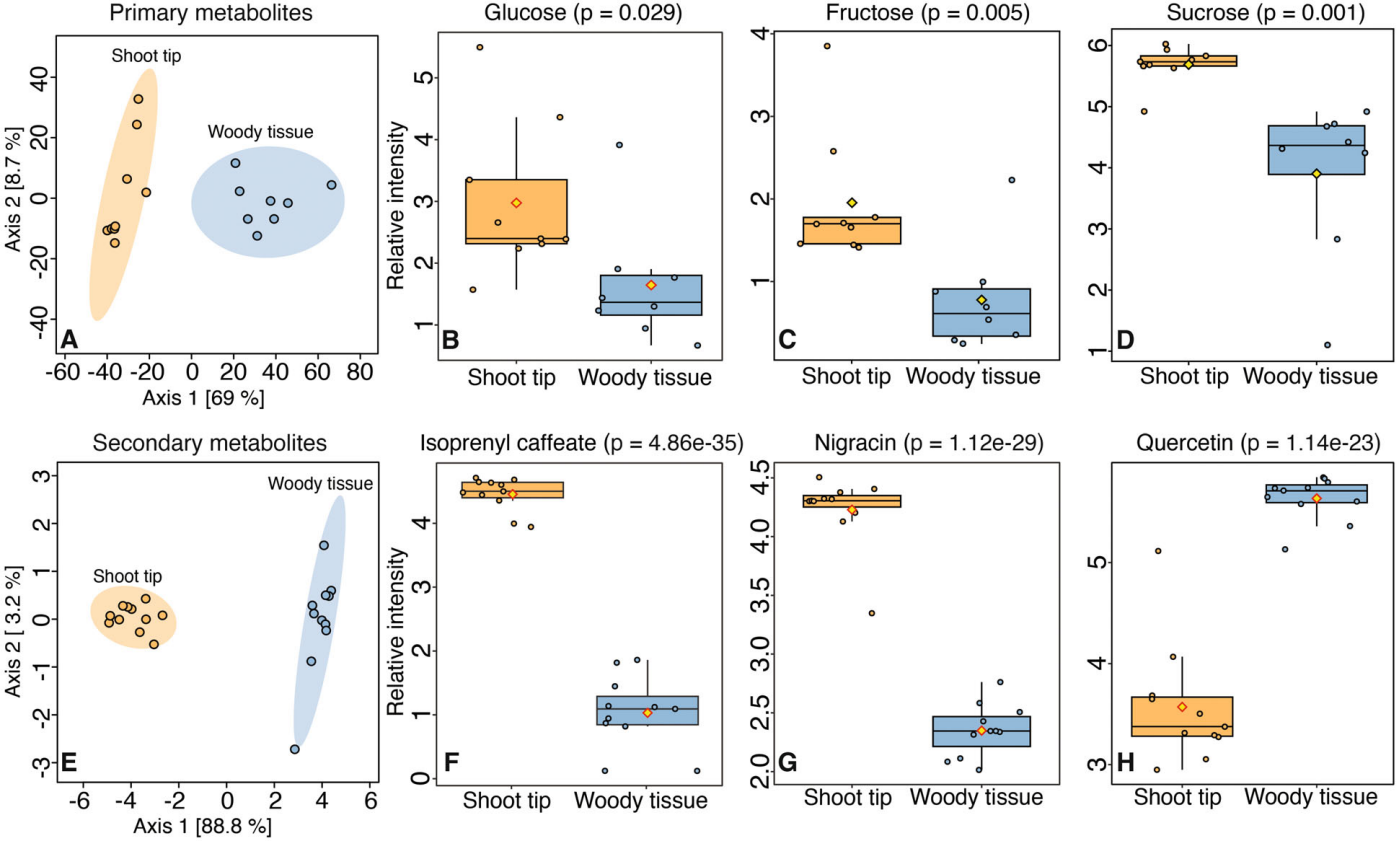
Abundant metabolites in the wood and shoot tips of *Populus nigra*. (A) PCA analysis of the relative abundances of major primary metabolites in shoot tips and stems. Relative abundances of (B) glucose, (C) fructose and (D) sucrose in both tissue types. (E) PCA of the relative abundances of major phenolic compounds in shoot tips and stems. Relative abundances of (F) isoprenyl caffeate, (G) nigracin and (H) quercetin in both tissue types. Primary metabolites were analysed by GC‐MS of sylilated samples. Secondary metabolites were analysed using LC‐MS. PCA was conducted using the standard settings in Metaboanalyst. Pairwise statistical comparisons were conducted using a two‐tailed *t* test. N = 8–10.

Significantly higher relative concentrations of sugars, fatty acids and organic acids were recovered from the shoot tip compared to the woody tissue, based on the GC‐MS analysis of silylated metabolites (Fig. 2A, Table S1). For example, there was on average a 2.5‐fold higher concentration of glucose (Fig. 2B), a 4.5‐fold higher concentration of fructose (Fig. 2C) and a 1.9‐fold higher concentration of sucrose (Fig. 2D) in the shoot tip relative to the wood (*P* < 0.05).

The distribution of phenolics, the most abundant secondary metabolites in poplar, also differed significantly in the shoot tip compared to the wood (Fig. 1E, Table S2). Phenolic acid derivatives, such as isoprenyl caffeate (Fig. 1F), and salicinoids, such as nigracin (Fig. 1G), were on average 5‐fold more abundant in the shoot tip relative to the wood (*P* < 0.01). On the other hand, flavonoids, such as quercetin (Fig. 6H), accumulated to 4‐fold higher concentrations in the woody tissue relative to the shoot tip (*P* < 0.01).

### Bioinformatic and statistical analyses of bacterial biodiversity data

**Fig. 3:**
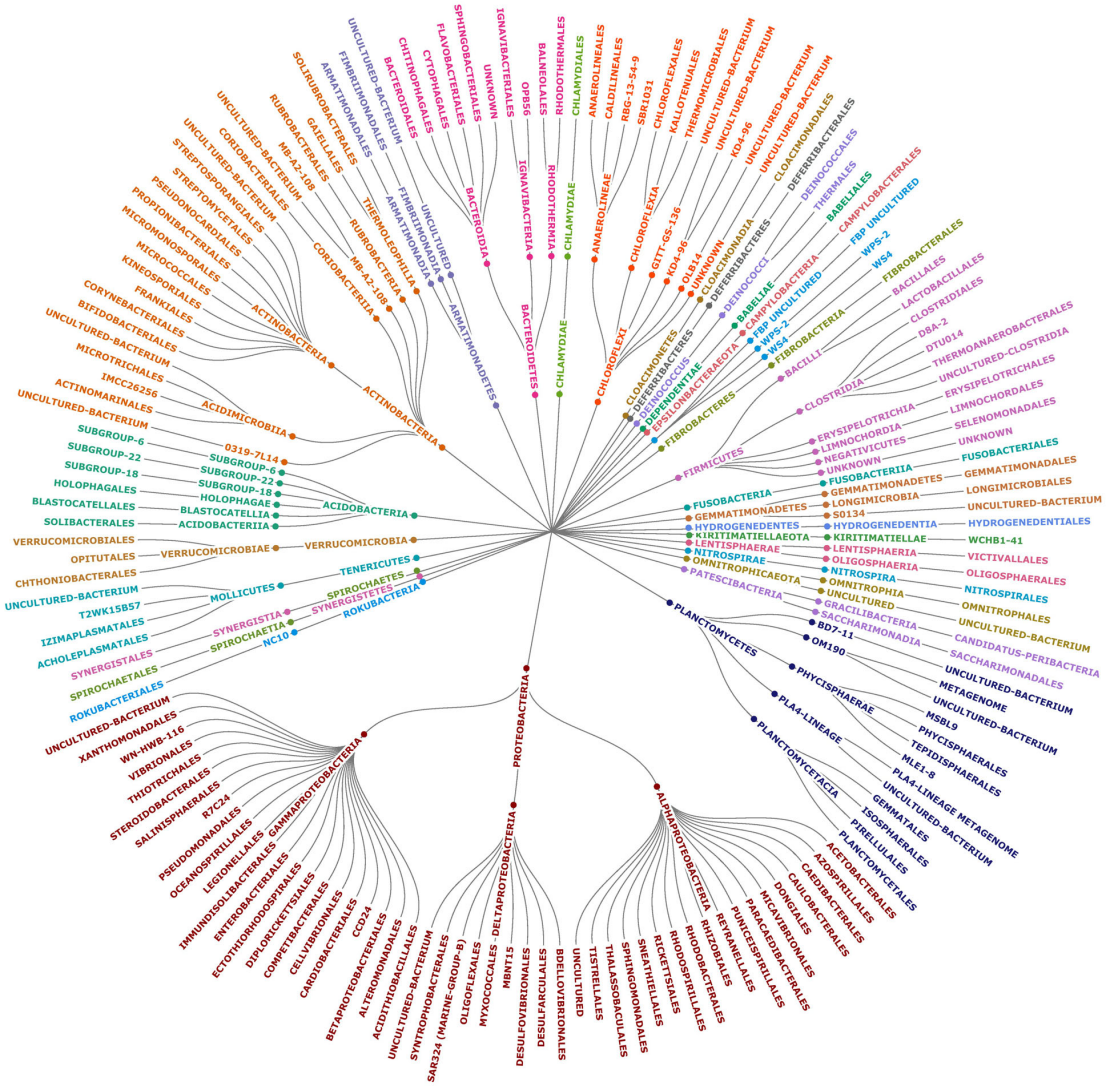
Bacterial orders associated with shoot tips and woody tissue of the *Populus nigra* cuttings.

The high‐throughput sequencing of 24 DNA samples yielded 3,040,030 raw reads. After merging the paired‐end reads using BBMerge, 2,408,779 (79.23%) were imported into Qiime2. Following filtering these raw reads using the “q2‐dada2” algorithm, 2,127,509 reads with an average length of 285 bp were used for downstream analyses. Following the exclusion of OTUs originating from chloroplasts and mitochondria, the resulting OTU table contained 373,791 reads. This corresponded to a total of 821 OTUs, identified at the species level whenever possible (Table S1). However, taxonomic levels above the order level were considered unreliable in this study. Consequently, we collapsed the OTU table to the order level that had 171 OTUs corresponding to 113 well‐defined orders, and numerous phylotypes, such as MB‐A2‐108, OPB56, RBG‐13‐54‐9, SBR1031, OLB14, KD4‐96 and many others (Fig. 3). This OTU table was used for all subsequent analyses.

**Fig. 4:**
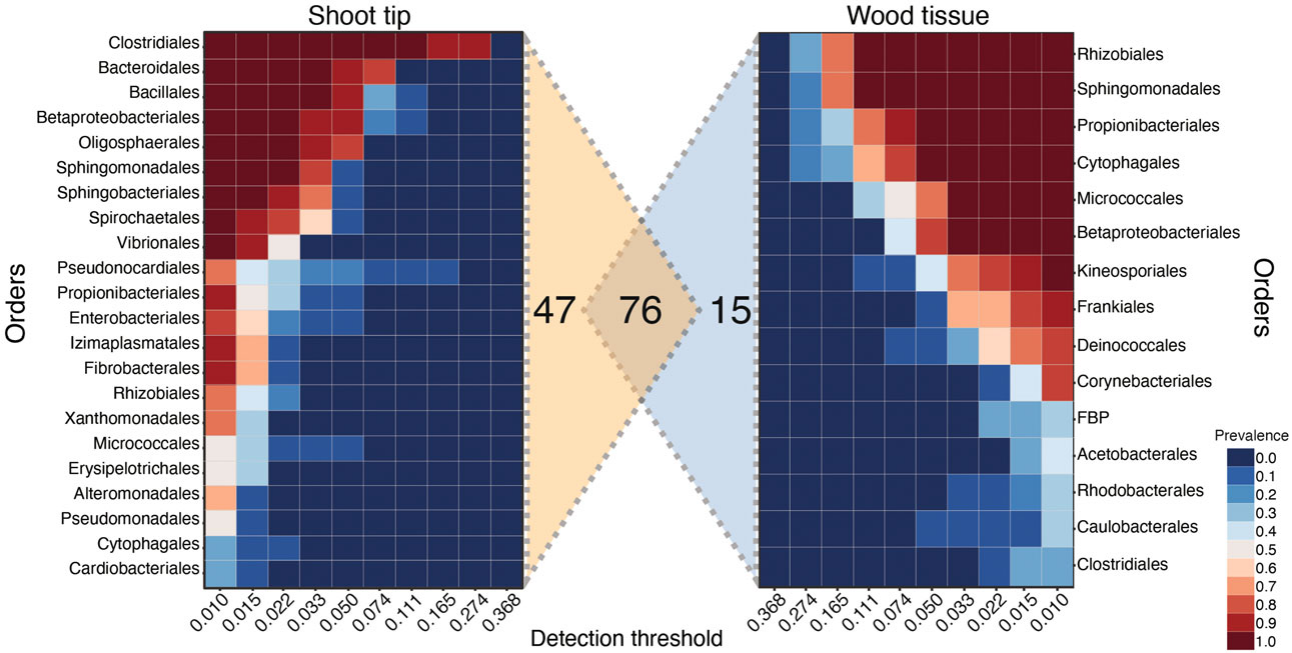
The abundance of bacterial orders associated with shoot tips and woody tissue of the *Populus nigra* branches. The heat map was constructed using the ‘core microbiome’ function of MicrobiomeAnalyst. Shared and unique bacterial orders between the two sample types are represented using a Venn diagram.

At the shoot tip, 34,807 reads corresponding to 99 well‐defined bacterial orders and 24 phylotypes were identified (Table S1). Clostridiales, Bacteriodales, Bacillales, Betaproteobacteriales, and Oligosphaerales were the five most dominant bacterial orders at the shoot tip (Fig. 4). In the wood tissue, 338,984 reads were detected, which corresponded to 81 well‐defined bacterial orders and 11 phylotypes (Table S1). Rhizobiales, Sphingomonadales, Propionibacteriales, Cytophagales, and Micrococcales were the most prevalent orders in the woody tissue (Fig. 4). Among these, 76 orders and phylotypes were shared between the two tissue types, while 47 were exclusive to shoot tips and 15 to woody tissues (Fig. 4).

**Fig. 5:**
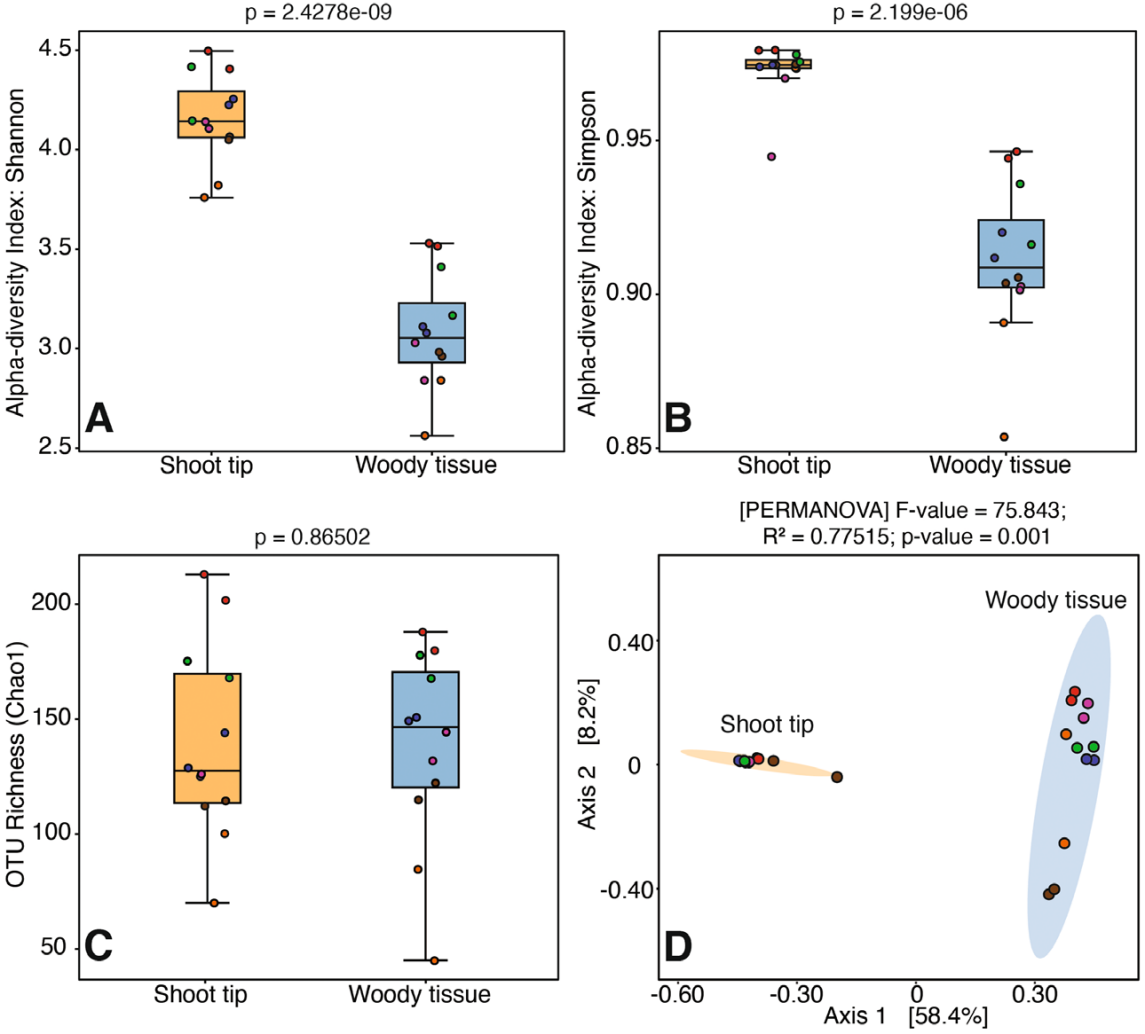
Plots comparing diversity indices and OTU richness between the two sample types, shoot tip and woody tissue, of *Populus nigra*. (A) Shannon index, (B) Simpson index, (C) OTU richness (Chao1), and (D) Principal Coordinates Analysis (PCoA). Data points with similar colours indicate samples originating from the same *P. nigra* cutting.

The alpha diversity indices, such as Shannon and Simpson, were significantly different for the two sample types: woody tissue and shoot tip (Shannon: *P* < 0.05, Simpson: *P* < 0.05; Fig. 5A, B). However, the OTU richness (chao1) was not significant between the sample types (*P* > 0.05; Fig. 5C). In these analyses, the samples originating from the same tree grouped close to each other (Fig. 5A–C). The data points in the PCoA plot clustered by sample type, which was validated by PERMANOVA (*F*‐value = 29.77, *R* ^2^ = 0.575, *P* = 0.001; Fig. 5D).

### Functional annotation of bacterial biodiversity data

**Fig. 6:**
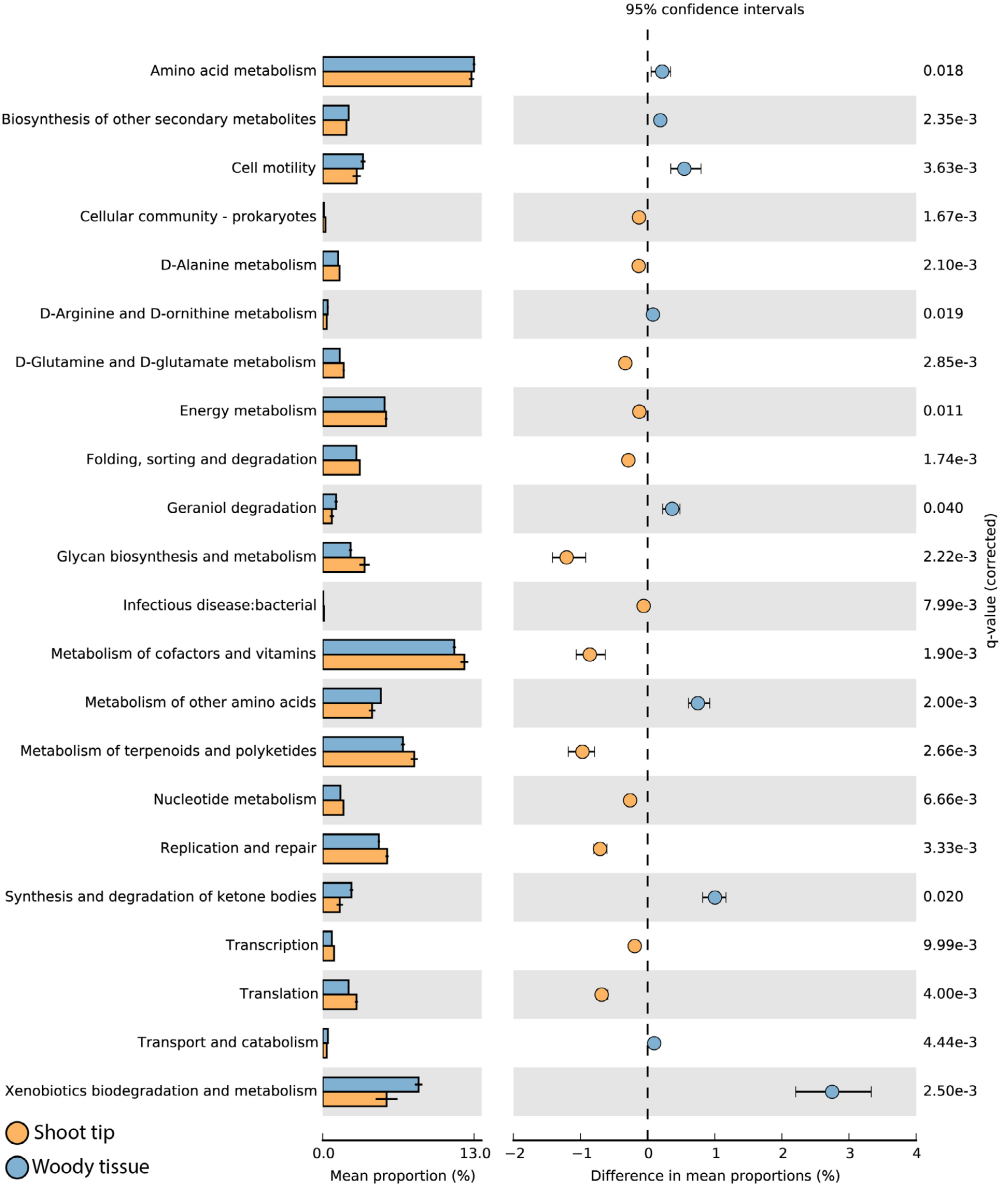
Bar plot showing the mean proportion percentage of 22 predicted functional categories within bacterial communities associated with the shoot tips and woody tissues of *Populus nigra*. Points indicate differences between shoot tips and woody tissues, with corresponding q‐values from White's non‐parametric *t*‐test.

Among the associated KEGG pathways, 40 were identified within our samples (Table S4). Out of these, 28 pathways displayed statistical significance (*P* < 0.05). Among these, six pathways were excluded due to their inconsistency with bacterial metabolic pathways, such as cardiovascular disease, neurodegenerative disease, and others (Table S4). Among the remaining 22 pathways, nine were significant within the woody tissue samples, while the others were significant within the shoot tip samples (Fig. 6). Functional pathways detected within the woody tissue encompassed amino acid metabolism, cell mortality, D‐arginine and D‐ornithine metabolism, ketone body synthesis and degradation, xenobiotic biodegradation and metabolism, among several others (Fig. 6). Fundamental metabolic functions, such as transcription, translation, replication and repair, nucleotide metabolism, energy metabolism, processes involving folding, sorting and degradation, co‐factors and vitamin metabolism, were predominant among the shoot tip samples (Fig. 6).

## DISCUSSION

In this study, we compared the metabolite profiles, bacterial biodiversity and functions associated with woody and green apical tissues of *P. nigra*. Our findings confirmed our hypotheses. Biochemical analyses showed significant enrichment of sugars, fatty acids, organic acids and phenolic acid derivatives in shoot tips, while flavonoids were higher in woody tissue. Bioinformatic and statistical analyses of sequence data revealed significant differences in alpha diversity indices (Shannon and Simpson) between the tissue types, although richness (chao1) did not differ. The PCoA plot showed data points clustering by tissue type, validated by PERMANOVA. Consequently, the functional diversity of the bacterial community also varied, with woody tissue showing higher levels of bacterial amino acid metabolism, D‐arginine and D‐ornithine metabolism, ketone body synthesis and degradation and xenobiotic biodegradation, while shoot tips had higher levels of fundamental bacterial metabolic processes such as transcription, translation, replication, repair and nucleotide metabolism.

### Host metabolite composition affects the bacterial biodiversity

Photosynthates are translocated from source tissues, such as fully developed leaves, to sink tissues, which are the actively growing parts of the plant (Knoblauch & Peters 2013). This process results in higher concentrations of sugars, fatty acids, and organic acids in shoot tips. As seen in this study, these elevated concentrations of primary metabolites play a pivotal role in fostering a diverse bacterial community by providing abundant resources for growth and metabolic activities. Conversely, high concentrations of phenolic acid derivatives and salicinoids in the shoot tip may play a potential role in safeguarding the actively growing tissues against potential pathogens or herbivores (Bednarek 2012; Paetz *et al*. 2016; Zaynab *et al*. 2018). The low abundance of bacterial reads coupled with the high species abundance in *P. nigra* shoot tips leads us to propose that the bacteria colonizing the shoot tips may comprise r‐selected species (Andrews & Harris 1986). R‐selected species are characterized by their rapid growth in resource‐rich environments, and reduced fitness under unfavourable conditions, such as the high levels of secondary metabolites that we detected in the shoot tips.

In the woody tissue, a lower concentration of photosynthates was detected, along with higher levels of flavonoids. However, compared to shoot tips, we detected 10‐fold more bacterial reads from the woody tissue of *P. nigra*. We thus hypothesize that bacteria inhabiting the woody tissue are mostly K‐selected species (Andrews & Harris 1986), known for their slower growth rate but higher fitness in resource‐poor environments. It is even conceivable, that the bacteria in the woody tissue can use flavonoids as carbon sources via the quercetin dioxygenase (Chen *et al*. 2019a, 2019b; Wang *et al*. 2022) or the ketoadipate pathways (Li *et al*. 2010), which have been detected in bacteria (Bowater *et al*. 2004).

### Bacterial biodiversity and community composition differed in the shoot tip and wood of *P. nigra*

In this study, the bacterial biodiversity detected in the tissue types from *P. nigra* underscore the dynamic nature of plant‐associated microbiomes. The alpha diversity indices reflected significant differences between shoot tip and woody tissue samples, indicating varied evenness of bacterial communities. The higher alpha diversity observed in the shoot tips could be attributed to its nutrient‐rich and rapidly growing nature, which provides a favourable yet challenging environment for a more diverse bacterial community. Moreover, in these analyses, the samples emerging from the same branch of *P. nigra* grouped closely, indicating an additional influence of plant genetics on bacterial biodiversity (Brown *et al*. 2020; Brachi *et al*. 2022). However, based on our results, the plant tissue and associated metabolites most likely had a greater influence on bacterial biodiversity. This is supported by the PCoA plot, which showed a distinct clustering of samples based on tissue types and metabolites.

The distinct microbial orders detected in shoot tip and woody tissue samples highlight the ecological partitioning between these regions (Ottesen *et al*. 2013; Junker & Keller 2015; Massoni *et al*. 2020). The shoot tip exhibited a dominance of orders associated with rapid growth, such as Clostridiales, Bacteroidales and Bacillales. A substantial diversity of bacteria from these orders are anaerobes, which are often linked to fermentation. The high prevalence of potential anaerobic fermenters in the shoot tip of *P. nigra* indicates that the sugars that are translocated by the plant to this region to support growth are substrates for these bacteria.

In contrast, the woody tissue was enriched with diazotrophic bacterial orders, namely Betaproteobacteriales, Frankiales, Rhizobiales and Sphingomonadales. Many known diazotrophs fix atmospheric dinitrogen to form ammonia that is used by plants for metabolic functioning (Fürnkranz *et al*. 2008; Rico *et al*. 2014; Kuypers *et al*. 2018). This is facilitated by the nitrogenase enzyme encoded by *nif* genes (Rico *et al*. 2014). In the case of non‐nodulating plants like poplar, which can grow in nutrient‐poor environments, a symbiotic relationship is sometimes established with endophytes, enabling these plants to acquire essential nitrogen (Doty *et al*. 2009; Doty *et al*. 2016). Thus, in our study, these diazotrophic bacteria detected from the wood may aid poplar in nitrogen fixation, as has been previously observed by Doty *et al*. (2016) in wild *P. trichocarpa* trees.

The diazotrophic bacteria within the wood and shoot tip of *P. nigra* likely originate from the soil in which the original poplar trees were growing and from which cuttings were harvested. This is because soil houses an enormous biodiversity of bacteria, forming the initial inoculum for the root microbiome. This was demonstrated for *Arabidopsis thaliana* (Bulgarelli *et al*. 2012; Lundberg *et al*. 2012). Moreover, in *Vitis vinifera*, the bacterial communities of leaves, flowers, and fruits displayed a higher proportion of shared taxa with soil communities than with each other. This suggests that soil might act as a common bacterial reservoir for both belowground and aboveground plant microbiomes (Zarraonaindia *et al*. 2015), and that this microbiome was transferred from the mother tree to the cuttings rooted in sterile soil and water. However, to conclusively identify the origin of the diazotrophs we detected in poplar wood, further comprehensive studies are required.

### Metabolic functions of bacterial communities in shoot tips and wood differed significantly in the two tissue types

The insights gleaned from the metabolic functional predictions based on KEGG pathway analysis shed light on the potential roles of bacteria within distinct tissue types of *P. nigra*. Notably, the woody tissue exhibited enrichment in pathways linked to amino acid metabolism, cell mortality and xenobiotic biodegradation. These predictions suggest potential microbial wood degradation processes or defence mechanisms against toxic compounds. In contrast, the shoot tip displayed enrichment in primary metabolic functions like transcription, translation, and energy metabolism. These functions suggest a high growth and turnover rate of bacteria living in the actively developing shoot.

Our observations align with findings from prior studies that compared bacterial functions across various plant organs. For instance, in a study by Bai *et al*. (2015), a comparison of bacterial functions between *Arabidopsis* leaf and root microbiota revealed enrichment of ‘carbohydrate metabolism’ in leaves and soil relative to roots. Notably, ‘xenobiotic biodegradation and catabolism’ were enriched in roots compared to those from leaves. On the other hand, Zarraonaindia *et al*. (2015) showed that microbial xenobiotic biodegradation and metabolism were more represented in fruits compared to other plant organs in *Vitus vinifera*. It is well known, that grapes accumulate high levels of flavonoids, such as anthocyanidins and flavan‐3‐ols in their fruit (Gürbüz *et al*. 2007). Poplar stems in our study also accumulated higher concentrations of flavonoids compared to the shoot tips and showed enrichment in microbial xenobiotic biodegradation and metabolism pathways. Microbial species composition and their associated metabolic activities are thus closely linked to the chemical composition of the substrate.

## CONCLUSIONS

In conclusion, this study sheds light on the intricate relationship between plant metabolites and bacterial communities within distinct tissues of *P. nigra* saplings. The study highlights the significance of tissue‐specific metabolic profiles in influencing bacterial biodiversity and community composition. These findings contribute to our understanding of the complex interactions between plants and their associated microbiomes, paving the way for further investigations into the mechanisms driving these relationships.

Further research could focus on characterizing specific bacterial taxa associated with these metabolites and understanding their functional contributions to plant health, growth, and defence. Additionally, investigating the effects of environmental variables such as light intensity, temperature, and soil conditions on these tissue‐specific microbial communities could provide a more comprehensive understanding of their dynamics.

## AUTHOR CONTRIBUTIONS

Conceptualization: A Hammerbacher, T Bose; Methodology: T Bose, A Hammerbacher, TG Mahomed, KC Mbatha, JC Joubert; Writing – original draft preparation: T Bose; Writing – review and editing: T Bose, A Hammerbacher, TG Mahomed, KC Mbatha, JC Joubert; Funding: A Hammerbacher. All authors have read and agreed to the published version of the manuscript.

## FUNDING INFORMATION

Max Planck Society provided funding for the Illumina high‐throughput sequencing used in this study. We acknowledge additional funding from the National Research Foundation of South Africa, Forestry South Africa, Tree Protection Cooperative Programme (FABI), and the University of Pretoria.

## Supporting information


**Table S1.** List of high‐confidence primary metabolites detected from shoot tips and wood of *Populus nigra* using gas chromatography–mass spectrometry.
**Table S2.** List of phenolics detected from shoot tips and woody tissues of *Populus nigra*.
**Table S3.** List of bacterial OTUs detected from shoot tips and woody tissues *Populus nigra*.
**Table S4.** List of functional pathways detected from *Populus nigra* samples using PICRUSt2. Statistical significance was calculated using STAMP.

## Data Availability

High‐throughput sequence data generated in this study are available through the NCBI Sequence Read Archive under the accession number PRJNA925423. Biochemistry data used in this study are available from the corresponding authors upon request.
